# One-year efficacy and safety of routine prasugrel in patients with acute coronary syndromes treated with percutaneous coronary intervention: results of the prospective rijnmond collective cardiology research study

**DOI:** 10.1007/s12471-018-1126-0

**Published:** 2018-06-21

**Authors:** T. Yetgin, E. Boersma, P. C. Smits, A. G. de Vries, E. Huijskens, F. Zijlstra, M. M. J. M. van der Linden, R. J. M. van Geuns, CCR Study Investigators

**Affiliations:** 1 Department of Cardiology, Thorax center, Erasmus MC, Rotterdam, The Netherlands; 2 Department of Cardiology, Maasstad Ziekenhuis, Rotterdam, The Netherlands; 3 Department of Cardiology, Albert Schweitzer Ziekenhuis, Dordrecht, The Netherlands; 4 Department of Cardiology, Franciscus Gasthuis & Vlietland, Schiedam, The Netherlands; 5 Department of Cardiology, Radboudumc, Nijmegen, The Netherlands

**Keywords:** Acute coronary syndrome, Percutaneous coronary intervention, Prasugrel

## Abstract

**Objective:**

To investigate 1‑year outcomes with routine prasugrel treatment after acute coronary syndrome (ACS) in a large-scale registry.

**Methods:**

The Rijnmond Collective Cardiology Research registry is a prospective, observational study that enrolled 4,258 consecutive ACS patients treated with percutaneous coronary intervention (PCI) with 1‑year follow-up. Patients received prasugrel as first-choice antiplatelet agent, except for increased bleeding risk patients in which clopidogrel was recommended. Events were validated by an independent clinical endpoint committee.

**Results:**

A total number of 2,677 patients received prasugrel at discharge after the index event. Eighty-one percent of the target population was on prasugrel treatment at hospital discharge. At 1 year, the primary endpoint, a composite of all-cause mortality and myocardial infarction, occurred in 2.4% of patients receiving prasugrel. All-cause mortality occurred in 1.0%, myocardial infarction in 1.5%, target-vessel revascularisation in 3.1%, stent thrombosis in 0.6%, and stroke in 0.5% of the patients treated with prasugrel. Thrombolysis in Myocardial Infarction defined major bleeding episodes not related to coronary artery bypass grafting were observed in 1.4% of patients receiving prasugrel.

**Conclusions:**

In routine practice, a tailored approach of prasugrel prescription in ACS patients undergoing PCI, resulted in low ischaemic and low bleeding rates up to 1 year post PCI.

**Electronic supplementary material:**

The online version of this article (10.1007/s12471-018-1126-0) contains supplementary material, which is available to authorized users.

## What’s new?

- Real-world data with current potent antiplatelet therapies is limited to low frequency use.
- A single antiplatelet therapy protocol was successfully implemented in 8 non-PCI-capable and 3 PCI-capable collaborating hospitals for all ACS patients.
- This resulted in a high penetration of current potent antiplatelet therapies with a low prescription rate for non-recommended or contra-indicated patients
- Within the high frequency use real-world data population no excess major bleeding events were observed.

## Introduction

Current European and North-American guidelines provide a Class I recommendation for the administration of dual antiplatelet therapy to patients presenting with acute coronary syndrome (ACS) who are undergoing percutaneous coronary intervention (PCI), consisting of aspirin to inhibit platelet thromboxane production and one of the newer P2Y12 receptor antagonists, such as prasugrel, to prevent secondary platelet activation [1, 2]. This recommendation was introduced following large classic randomised trials [3, 4].

As with every novel pharmacological principle, documentation of real-life contemporary results is of utmost importance, especially when considering that baseline and procedural characteristics in clinical trial participants differ considerably from those in non-participants along with a better survival observed in trial participants [5]. In this sense longitudinal clinical registries that provide information on the effectiveness and safety in real-world patient populations are of utmost importance. Accordingly, our aim was to study the introduction of prasugrel into contemporary practice to understand the appropriateness of its use. Hence, we aimed to observe treatment patterns and 1‑year outcomes associated with routine prasugrel treatment using a tailored strategy in PCI-treated ACS patients in the large-scale prospective Rijnmond Collective Cardiology Research (CCR) study.

## Methods

### Study design and population

Full details of the CCR study rationale and methodology (*Dutch Trial Register identifier: NTR3704*) have been reported elsewhere [6]. In brief, the CCR study was a prospective, multicentre, observational registry of management practices and outcome of ACS up to 12 months post-discharge involving three high-volume centres with PCI capability and eight non-PCI centres in the Rijnmond region in the Netherlands (Appendix A). The CCR study was initiated in August 2011, after the Guideline Committee of the participating network updated the treatment guidelines to include prasugrel as the first-line treatment option for antiplatelet therapy in PCI patients. A maintenance dose of 10 mg prasugrel was preferred, while a maintenance dose of 75 mg clopidogrel was recommended for patients with prior stroke or transient ischaemic attack (TIA) and 5 mg prasugrel for patients over 75 years or weighing less than 60 kg following the European label for prasugrel where 75 mg clopidogrel served as an alternative when 5 mg prasugrel was not available. To avoid the off-label use of prasugrel, clopidogrel was recommended in patients with a high clinical bleeding risk. We completed the enrolment in June 2013. No treatment intervention was directed by protocol in the CCR study. Therefore, the treating physicians made all treatment decisions in accordance with practice guideline recommendations and local standards of care and practice.

Patients were not subjected to special treatments or diagnostic test or imposed to any mode of behaviour for the purpose of this study, other than standard treatment. Therefore, according to Dutch law, we did not require written informed consent for a patient to be enrolled in this study. This study was conducted according to the Privacy Policy of the Erasmus MC and according to the Erasmus MC regulations for the appropriate use of data in patient-oriented research. It was also approved by the regional ethics committee.

### Data collection and event validation

Patient characteristics, clinical features, angiographic and procedural details, and in-hospital outcomes were abstracted from the medical chart per routine and entered into a secure web-based centralised database. After the index hospitalisation, patients were routinely followed up at 1 month and 12 months at the outpatient clinics of the enrolling sites where medication adherence was checked. Dedicated study staff visited the individual sites for monitoring and all events were validated by an independent clinical endpoint committee [6].

### Study endpoints

The primary endpoint for the current study was the composite of all-cause mortality and non-fatal myocardial infarction (MI) for hospital survivors discharged on long-term prasugrel therapy. Secondary efficacy endpoints included the composite of cardiovascular death, non-fatal MI and target vessel revascularisation defined as major adverse cardiac events (MACE), stroke, stent thrombosis (ST) according to definite or probable Academic Research Consortium definitions [7], and all individual components of the composite endpoints, as previously defined [6]. Safety endpoints included thrombolysis in myocardial infarction (TIMI) major and minor bleeding events that were unrelated to coronary artery bypass grafting (CABG) after index PCI, as defined per TRITON-TIMI 38 criteria [4].

### Statistical analysis

Continuous variables are summarised as mean ± standard deviation (SD) or median with interquartile range (IQR), depending on the distribution pattern. We compared the continuous variables using the Student’s *t*-test (normal distribution) or Mann-Whitney U‑test (non-normal distribution) as appropriate. Categorical variables are summarised as frequencies and percentages and were compared using the chi-square test. Clinical outcomes are presented as the cumulative incidence based on the Kaplan-Meier method (%). Patients lost to follow-up were considered at risk until the date of last contact, at which time point they were censored. Statistical tests were two-sided and a *p*-value < 0.05 was considered statistically significant. Computations were performed using SAS version 9.2 (SAS Institute, Cary, NC, USA).

## Results

### Demographic characteristics

**Fig. 1:**
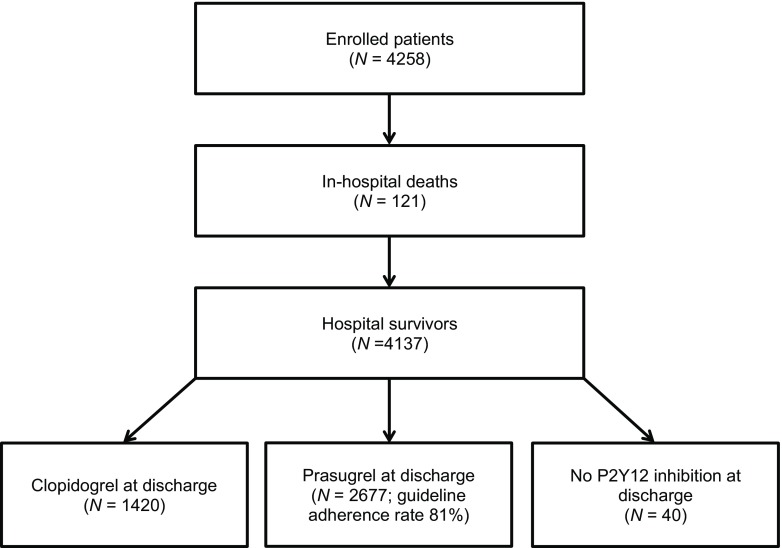
Study flow chart

During the enrolment period a number of 4,258 consecutive ACS patients underwent PCI according to the PCI databases of the 3 referral centres and were enrolled in the regional registry (Fig. 1). Demographic and clinical characteristics of the total cohort are listed in Tab. 1. During initial hospitalisation, 121 patients died and were not part of the study cohort. Characteristics of these patients are described in the online supplementary Tab. X1, with a high frequency of ST-elevation myocardial infarction (STEMI) presentation followed by either ongoing cardiogenic shock or severe neurological damage after out-of-hospital cardiac arrest. Of the hospital survivors 27.2% (*n* = 1,124) had at least one characteristic of increased bleeding risk and an additional 183 patients were on vitamin K antagonists (details in the online supplementary Tab. X2). Therefore, the potential prasugrel cohort was reduced to 2,830 patients of whom 2,282 were on target therapy at discharge (81%) (Fig. 1). As 395 patients of the increased risk group were still treated with 5 or 10 mg prasugrel, the total study cohort comprised 2,677 patients, 65% of all patients (Fig. 1). Demographic and clinical characteristics are also listed in Tab. 1 and procedural details of the study cohort are listed in Tab. 2. Patients discharged on prasugrel were younger and had less clinical comorbidities compared with patients discharged on clopidogrel. The median age of the study cohort was 60.0 years, 23% were female and 14% had diabetes. Patients ≥ 75 years of age or weighing <60 kg, comprising subgroups in whom cautionary use of prasugrel is advised, totalled 7.8 and 2.9% of the study cohort, respectively. Prasugrel prescription in patients with prior stroke or TIA was very low (*n* = 25, <1%). Half of the study cohort (50%) presented with STEMI, 35% with non-ST-elevation myocardial infarction (NSTEMI), and 15% with unstable angina. In 56.2% of the population radial access was preferred.

**Table 1:** Demographic and clinical characteristics

	All patients (*N* *=* 4,258)	Patients receiving clopidogrel at discharge (*N* *=* 1,420)	Patients receiving prasugrel at discharge (*N* *=* 2,677)
*Demographics*			
Age, year	64 (54, 73)	73 (62, 79)	60 (52, 68)
Age ≥ 75	21.5	45.1	7.8
Woman	27.5	35.7	23.0
Weight, kg	80 (73, 91)	80 (70, 90)	82 (74, 93)
Weight <60 kg	4.0	5.9	2.9
*Cardiovascular risk factors*			
Diabetes mellitus	17.0	21.5	14.0
Hypertension	51.6	61.9	45.6
Hypercholesterolaemia	37.6	41.9	35.1
Current smoking	36.0	21.9	43.6
Family history of CAD	44.3	39.5	47.6
*Cardiovascular disease history*			
MI	18.3	24.5	15.0
PCI	18.0	25.1	14.4
CABG	5.6	10.3	3.2
Stroke or TIA	7.3	18.4	0.9
Peripheral artery disease	7.2	10.7	4.8
Congestive heart failure	3.8	5.6	2.7
*Laboratory findings*			
Creatinine, µmol/l	80 (69, 95)	83 (70, 101)	78 (68, 91)
Haemoglobin, mmol/l	8.9 (8.2, 9.5)	8.7 (7.9, 9.3)	9.0 (8.4, 9.6)
Thrombocytes, •10^9^/l	234 (195, 277)	233 (194, 277)	234 (196, 278)
Positive (>13 ng/l) high-sensitivity troponin test during admission	85.4	79.8	87.9
*Presentation*			
Admission diagnosis			
– Unstable angina	19.4	28.4	15.2
– NSTEMI	36.9	43.3	34.6
– STEMI	43.8	28.3	50.1
*Left ventricular function* ^a^			
– Normal (≥50%)	66.1	63.2	68.2
– Moderate (30–49%)	29.4	30.5	28.7
– Poor (<30%)	4.5	6.3	3.1

Continuous data are presented as median (25th, 75th percentile), and categorical data are presented as percentages. All differences in characteristics between patients receiving clopidogrel at discharge and those receiving prasugrel at discharge were statistically significant (*p* < 0.001), except median thrombocytes (*p* = 0.36)

*CABG* coronary artery bypass grafting; *CAD* coronary artery disease; *IQR* interquartile range; *MI* myocardial infarction; *NSTEMI* non-ST-elevation myocardial infarction; *PCI* percutaneous coronary intervention; *SD* standard deviation; *STEMI* ST-elevation myocardial infarction; *TIA* transient ischaemic attack

^a^Available in 2,920 patients

**Table 2:** Treatments and procedures in patients receiving prasugrel at discharge

Timings and durations	***N*** = 2,677
Admission to PCI, days (by diagnosis)	
– Unstable angina	2.0 (0, 4.0)
– NSTEMI	1.0 (0, 4.0)
– STEMI	0 (0, 0)
Admission duration, days	4.0 (3.0, 6.0)
*Procedural characteristics*	
Access site	
– Femoral	39.1
– Radial	56.2
– Other	4.7
Treated vessel	
– Right coronary artery	37.1
– Left main	2.5
– Left anterior descending	46.8
– Left circumflex	25.2
– Arterial or saphenous grafts	1.3
Multivessel PCI during index procedure	13.5
*Periprocedural antithrombotic treatment*	
Aspirin	88.3
Clopidogrel	12.6
Prasugrel	78.5
Clopidogrel and prasugrel	0.7
Glycoprotein IIb/IIIa inhibitor	17.0
Unfractionated heparin^a^	100
Low-molecular-weight heparin	2.7

Continuous data are presented as median (25th, 75th percentile), and categorical data are presented as percentages

*NSTEMI* non-ST-elevation myocardial infarction; *PCI* percutaneous coronary intervention; *STEMI* ST-elevation myocardial infarction

^a^We did not record heparin use in individual patients, but heparin is used during PCI procedures in patients with acute coronary syndrome as per treatment protocol in the regional PCI centres

### Pharmacological treatment

The discharge prescription rates and medication use at follow-up in the study cohort are listed in the online supplementary Tab. X3. A prasugrel maintenance dose of 10 mg was instituted in 93.3% of patients at discharge, whereas 6.7% of patients received a maintenance dose of 5 mg, which declined to 5.4% at 1‑year follow-up. The prescription rates of a maintenance dose of 10 mg prasugrel at discharge in patients ≥ 75 years of age or weighing <60 kg were very low (2.4%) indicating a very high adherence to the regional protocol. Triple antithrombotic therapy with aspirin, prasugrel and a vitamin K antagonist was instituted in 3.3% of patients at discharge and declined to 1.8% at 1‑year follow-up.

### Outcomes

Clinical follow-up was available for 2,615 patients (97.7%) at one month and 2,443 patients (91.3%) at one year. Of note, all patients lost to follow-up for nonfatal endpoints were reported alive by municipal civil registries. The efficacy and safety endpoints at one month and one year are presented in Tab. 3. The primary combined endpoint of all-cause mortality or non-fatal MI occurred in 0.8% of patients at one month and in 2.4% at one year. The incidence of the individual endpoints were very low at one year, with target vessel revascularisation comprising the most frequently observed individual endpoint with a 3.1% event rate. TIMI major bleeding events unrelated to CABG occurred in 20 (0.8%) patients at one month and in 1.4% at one year. The most common types of TIMI major bleeding events in the course of one month were bleeding at vascular access sites (*n* = 7; 35% of all bleeding events), gastrointestinal haemorrhage (*n* = 3), intracranial haemorrhage (*n* = 2) and other (*n* = 8). In the course of one year, 17 additional patients experienced a TIMI major bleeding event. One-year efficacy and safety data in selected strata are presented in the online supplementary Tab. X4.

**Table 3:** Clinical outcomes in patients receiving prasugrel at discharge

	In-hospital	1 month^a^	1 year^b^
*Composite efficacy outcomes*			
All-cause death or MI (primary endpoint)	13 (0.5)	22 (0.8)	64 (2.4)
Cardiovascular death, MI or TVR	23 (0.9)	41 (1.5)	119 (4.4)
*Single efficacy outcomes*			
All-cause death	–	4 (0.2)	28 (1.0)
Cardiovascular death	–	4 (0.2)	17 (0.6)
MI	13 (0.5)	19 (0.7)	39 (1.5)
Stent thrombosis	5 (0.2)	12 (0.4)	16 (0.6)
– Definite	5	11	13
– Probable	0	1	1
– Possible	0	0	2
TLR	10 (0.4)	17 (0.6)	44 (1.6)
TVR	16 (0.6)	30 (1.1)	83 (3.1)
CABG	2 (0.1)	6 (0.2)	22 (0.8)
Stroke	3 (0.1)	8 (0.3)	13 (0.5)
*Safety outcome*			
Non-CABG TIMI major bleeding	14 (0.5)	20 (0.8)	37 (1.4)
– Access site	5	7	10
– Gastrointestinal	2	3	8
– Intracranial	0	2	6
– Other	7	8	13

Data represent the number of patients with at least one of the respective outcomes at 1 month and 1 year, respectively, and the corresponding cumulative incidence based on the Kaplan-Meier method (%)

*CABG* coronary artery bypass grafting; *MI* myocardial infarction; *TIMI* thrombolysis in myocardial infarction*; TLR* target lesion revascularisation; *TVR* target vessel revascularisation

^a^30 days

^b^400 days

## Discussion

In this large-scale registry, 81% of all consecutive ACS patients treated with PCI who were eligible for a newer and more potent guideline-recommended P2Y12 receptor antagonist were discharged on prasugrel. These patients exhibited low rates of ischaemic events, including overall mortality post discharge, and low rates of major bleeding events in routine contemporary practice. These data provide important evidence with regard to the efficacy and safety of real-world use of prasugrel as part of an antiplatelet strategy with a tailored approach.

Our findings are consistent with the TRITON-TIMI 38 trial in terms of efficacy of prasugrel, with an even better safety profile. In TRITON-TIMI 38, prasugrel reduced the incidence of the primary endpoint of cardiovascular death, MI, and stroke compared with clopidogrel (9.9% vs. 12.1%; *P* < 0.001) in ACS patients undergoing PCI, but it also increased the risk for non-CABG TIMI major bleeding, particularly among the elderly (≥75 years), as well as in those with low body weight (<60 kg), prior stroke or TIA [3]. We observed much lower rates of efficacy endpoints along with low rates of bleeding. These results may be explained, at least in part, by the low-risk profile of the current study population and demonstrate the efficacy of our tailored approach in routine clinical practice where potentially 66% of all ACS-PCI patients were on full-dose high-effective dual antiplatelet therapy. In fact, our results are much more in line with recent TRITON-TIMI 38 subanalyses [8] in which the benefit of prasugrel was maximised and the risk of adverse outcomes limited by excluding high-risk patients. Importantly, in-hospital deaths after the index procedure were not part of the study cohort (*N* = 121) and non-fatal major bleeding events among these patients were limited (*n* = 7, of which 3 on prasugrel), whereas in-hospital bleeding events in the study cohort were still included for the analysis. In this respect, the currently observed low mortality rate is partly explained by our focus on hospital survivors only, whereas hospital mortality was mainly driven by patients with out-of-hospital cardiac arrest or patients who presented with cardiogenic shock.

When we focus on bleeding events, we see that prasugrel pretreatment significantly increased bleeding complications in ACCOAST-PCI [9], although the incidence of major bleeding was low at 30 days (1.7 and 0.66% in the pretreatment and no-pretreatment groups, respectively). The incidence of major bleeding at 30 days in the CCR registry was 0.8%. This is perfectly in line with the results from the ACCOAST-PCI study, and remarkably close to the bleeding events in the no-pretreatment arm. In this regard we should mention the higher number of radial artery access (56%) in the CCR study versus 56% femoral approaches in the ACCOAST-PCI study [9]. A recent subanalysis of the ACCOAST-PCI study demonstrated that pretreatment, age, gender and procedural variables (femoral access) were independent predictors of TIMI major or minor bleeding in patients with NSTEMI, a finding in line with the current results [10].

Our results extend previous observations in registries of PCI-treated ACS patients [11, 12]. For instance, Damman et al. demonstrated low rates of in-hospital bleeding and 30-day mortality for prasugrel in an analysis of the SCAAR data [11]. Our data are also in keeping with the recent large-scale TRANSLATE-ACS registry demonstrating lower (unadjusted) MACE in patients receiving prasugrel (*n* = 3,123) versus clopidogrel (*n* = 8,846) [13]. In contrast, the current observational study was initiated after an update of the treatment guidelines to include prasugrel as the first-line treatment option for antiplatelet therapy. Based on the strategy in our study, 65% of patients were discharged on prasugrel versus 26% in the TRANSLATE-ACS registry. With the current strategy we were also able to limit inappropriate or non-recommended use of prasugrel in contrast to another national registry [14].

In the current registry, 33% of patients were discharged on clopidogrel. To a large extent, this observation can be explained by the tailored approach for the use of prasugrel in our network with an individual risk-benefit evaluation in patients with ACS who are undergoing PCI. This percentage could have been lower if the 5 mg dosage for patients with an increased bleeding risk would have been reimbursed by health insurers in the Netherlands. Nonetheless, the tailored protocol enabled physicians to adequately triage patients to prasugrel according to their baseline and/or bleeding risk, thereby optimising safety and outcomes, as demonstrated by the present low ischaemic and low bleeding event rates. Currently, prasugrel and ticagrelor are the recommended first-line agents in patients with ACS. We cannot reliably establish which drug is superior over the other on the basis of the current data [15]. For example, both agents are similarly effective during the first year after MI [16] and based on a recent meta-analysis of observational and randomised studies totalling 21,360 patients, prasugrel appeared to be equivalent or superior to ticagrelor in patients with ACS who are undergoing PCI at 1‑month follow-up [17].

Our findings should be considered in the context of the following potential limitations. Inherent for all single-arm registry data, final efficacy and safety versus other strategies cannot be claimed. A follow-up rate of 97.7% at one month and 91.3% at one year represents substantial completeness of our data. Yet is unlikely that we missed any deaths (or potential major bleeding events leading to death) through confirmation of municipal civil registries. Even though we may have missed additional endpoints, we believe this may not considerably impact the overall conclusions of our findings.

Nonetheless, our data provide an objective snapshot of the use of prasugrel in daily practice where patients are selected on known increased bleeding characteristics, affording valuable insights into treatment effectiveness and generalisability [18]. The adherence rate of 81% at discharge, the potential prasugrel cohort on target therapy as described in the results section, was based only on prescription data and follow-up, as well as on patient interviews at the outpatient clinics of the enrolling sites. Unfortunately, the design of the study did not allow us to collect information regarding reasons for discontinuation, interruption or disruption of prasugrel [19]. Furthermore, we did not collect information regarding treatment decisions such as the specific reasoning for initially selecting thienopyridine or switching to thienopyridine in-hospital. Nor did we collect any information regarding specific timings such as the exact time of thienopyridine loading or time from loading dose to starting angiography/PCI.

## Conclusions

The CCR study shows that with the tailored antiplatelet therapy protocol in PCI-treated ACS patients in our region patients discharged on prasugrel are predominantly younger with less clinical comorbidities and/or presented with STEMI compared with the patients discharged on clopidogrel. This tailored approach resulted in low rates of ischaemic events, including overall mortality, and no excess rates of major bleeding events for patients on prasugrel in routine practice up to one year post PCI compared with earlier randomised findings and observational data.

### Caption Electronic Supplementary Material

Table X1 Characteristics of patients with in-hospital death

Table X2 Frequency of high bleeding risk factors

Table X3 Pharmacological treatment in patients receiving prasugrel at discharge

Table X4 One-year* clinical outcomes in patients receiving prasugrel at discharge in selected strata

## Appendix A

### Participating centres

Albert Schweitzer Ziekenhuis, Dordrecht; Beatrix Ziekenhuis, Gorinchem; Erasmus Medisch Centrum, Rotterdam; Havenziekenhuis, Rotterdam; IJsselland Ziekenhuis, Rotterdam; Ikazia Ziekenhuis, Rotterdam; Maasstad Ziekenhuis, Rotterdam; Ruwaard van Putten Ziekenhuis, Spijkenisse; Sint Franciscus Gasthuis, Rotterdam; Van Weel-Bethesda Ziekenhuis, Dirksland; and Vlietland Ziekenhuis, Schiedam. All centres are located in the Netherlands.
