# Supplementary material for: One-year efficacy and safety of routine prasugrel in patients with acute coronary syndromes treated with percutaneous coronary intervention: results of the prospective rijnmond collective cardiology research study
Source: Neth Heart J. 2018 Jun 21;26(7-8):393–400. doi: 10.1007/s12471-018-1126-0 (PMC6046662; doi:10.1007/s12471-018-1126-0)
Supplement: Supplementary file 1 — Table X1 Characteristics of patients with in-hospital death [file 12471_2018_1126_MOESM1_ESM.docx]

**Online supplementary Table X1 Characteristics of patients with in-hospital death**

|  | **In-hospital death (*N=*121)** |
| --- | --- |
| *Demographics* |  |
| Age, year | 72 (64, 78) |
| Age ≥75 | 42.2 |
| Female | 28.1 |
| Weight, kg | 80 (70, 85) |
| Weight <60 kg * | 4.8 |
| *Cardiovascular risk factors* |  |
| Diabetes mellitus | 29.8 |
| Hypertension | 60.2 |
| Hypercholesterolaemia | 39.6 |
| Current smoking | 33.3 |
| Family history of CAD | 23.3 |
| *Cardiovascular disease history* |  |
| MI | 18.5 |
| PCI | 13.0 |
| CABG | 2.7 |
| Stroke or TIA | 15.0 |
| Peripheral artery disease | 19.8 |
| Congestive heart failure | 7.3 |
| *Admission diagnosis* |  |
| Unstable angina | 1.7 |
| NTEMI | 14.9 |
| STEMI | 83.5 |
| *Peri/postprocedural P2Y12 inhibitor* |  |
| Clopidogrel | 32.2 |
| Prasugrel | 32.2 |
| *Reason of death* |  |
| Cardiovascular death | 82.6 |
| TIMI major bleeding | 5.8 |
| Other | 11.6 |

*CABG* coronary artery bypass grafting, *CAD* coronary artery disease, *IQR* interquartile range, *MI* myocardial infarction, *NSTEMI* non-ST-elevation myocardial infarction, *PCI* percutaneous coronary intervention, *SD* standard deviation, *STEMI* ST-elevation myocardial infarction, *TIA* transient ischaemic attack, *TIMI* thrombolysis in myocardial infarction

Continuous data are presented as median (25^th^, 75^th^ percentile), and categorical data are presented as percentages.
