# Supplementary material for: One-year efficacy and safety of routine prasugrel in patients with acute coronary syndromes treated with percutaneous coronary intervention: results of the prospective rijnmond collective cardiology research study
Source: Neth Heart J. 2018 Jun 21;26(7-8):393–400. doi: 10.1007/s12471-018-1126-0 (PMC6046662; doi:10.1007/s12471-018-1126-0)
Supplement: Supplementary file 2 — Table X2 Frequency of high bleeding risk factors [file 12471_2018_1126_MOESM2_ESM.docx]

**Online supplementary Table X2 Frequency of high bleeding risk factors**

| *Demographics* | *N*=4137 |
| --- | --- |
| Age ≥75 | 865 (20.9%) |
| Weight <60 kg * | 132 (3,2%) |
| History of stroke or TIA | 293 (7.0%) |
| *Any of the above* | *1124 (27.2%)* |
| Vitamin K antagonist | 183 (4.4%) |

*TIA* transient ischaemic attack

Data are presented as numbers and percentages in brackets.
