# Supplementary material for: One-year efficacy and safety of routine prasugrel in patients with acute coronary syndromes treated with percutaneous coronary intervention: results of the prospective rijnmond collective cardiology research study
Source: Neth Heart J. 2018 Jun 21;26(7-8):393–400. doi: 10.1007/s12471-018-1126-0 (PMC6046662; doi:10.1007/s12471-018-1126-0)
Supplement: Supplementary file 3 — Table X3 Pharmacological treatment in patients receiving prasugrel at discharge [file 12471_2018_1126_MOESM3_ESM.docx]

**Supplementary Table X3 Pharmacological treatment in patients receiving prasugrel at discharge**

| **Medication** | **Discharge (*N*=2677)** | **1 month (*N*=2617)** | **1 year (*N*=2445)** |
| --- | --- | --- | --- |
| *Antiplatelet and antithrombic drugs* |  |  |  |
| Aspirin | 96.9 | 95.5 | 91.4 |
| P2Y12 inhibitor | 100 | 98.8 | 82.9 |
| Prasugrel | 100 | 96.0 | 78.2 |
| Maintenance dose of 5 mg * | 6.7 | 6.3 | 5.4 |
| Maintenance dose of 10 mg * | 93.3 | 93.7 | 94.6 |
| Age ≥75 † | 2.4 | 2.7 | 2.5 |
| Weight <60 kg † | 2.3 | 2.4 | 2.3 |
| Clopidogrel | 0 | 3.0 | 4.7 |
| Ticagrelor | 0 | 0 | 0.2 |
| Vitamin K antagonist | 5.5 | 6.1 | 6.8 |
| Aspirin, P2Y12 inhibitor and vitamin K antagonist |  |  |  |
| None | 0 | 0.6 | 2.9 |
| Mono | 0.9 | 1.7 | 14.9 |
| Double | 95.8 | 94.5 | 80.4 |
| Triple | 3.3 | 3.2 | 1.8 |
| *Other cardiovascular drugs* |  |  |  |
| ACE-I/ARB | 85.2 | 84.7 | 81.4 |
| Statin | 97.2 | 96.1 | 91.8 |
| Beta-blocker | 86.8 | 86.5 | 77.4 |
| Calcium channel blocker | 13.4 | 14.4 | 16.2 |
| Nitrate | 6.7 | 8.2 | 6.9 |
| Antiarrhythmic agent | 3.1 | 3.5 | 3.5 |

*ACE-I/ARB* angiotensin-converting enzyme inhibitor or angiotensin receptor blocker

* in patients who used prasugrel

† in patients who used prasugrel 10 mg

Data are presented as percentages.
