# Supplementary material for: One-year efficacy and safety of routine prasugrel in patients with acute coronary syndromes treated with percutaneous coronary intervention: results of the prospective rijnmond collective cardiology research study
Source: Neth Heart J. 2018 Jun 21;26(7-8):393–400. doi: 10.1007/s12471-018-1126-0 (PMC6046662; doi:10.1007/s12471-018-1126-0)
Supplement: Supplementary file 4 — Table X4 One-year* clinical outcomes in patients receiving prasugrel at discharge in selected strata [file 12471_2018_1126_MOESM4_ESM.docx]

**Supplementary Table X4 One-year* clinical outcomes in patients receiving prasugrel at discharge in selected strata**

| **Stratum** | **Patients** |  | **All-cause death or MI** | | |  | **CV death, MI or TVR** | | |  | | **TIMI major bleeding** | | | |  |
| --- | --- | --- | --- | --- | --- | --- | --- | --- | --- | --- | --- | --- | --- | --- | --- | --- |
|  |  |  | ***N*** | **(%)** | ***p*-value †** |  | ***N*** | **(%)** | ***p*-value †** | |  | | ***N*** | **(%)** | ***p*-value †** | |
| Age |  |  |  |  | <0.001 |  |  |  | 0.005 | |  | |  |  | 0.517 | |
| <75 years | 2469 |  | 50 | (2.2) |  |  | 102 | (4.7) |  | |  | | 33 | (1.5) |  | |
| ≥75 years | 208 |  | 14 | (7.0) |  |  | 17 | (8.5) |  | |  | | 4 | (1.9) |  | |
| Sex |  |  |  |  | 0.941 |  |  |  | 0.456 | |  | |  |  | 0.002 | |
| Female | 615 |  | 15 | (2.6) |  |  | 25 | (4.4) |  | |  | | 18 | (3.0) |  | |
| Male | 2062 |  | 49 | (2.6) |  |  | 94 | (5.2) |  | |  | | 19 | (1.0) |  | |
| Weight |  |  |  |  | 0.035 |  |  |  | 0.961 | |  | |  |  | 0.803 | |
| <60 kg | 2042 |  | 43 | (2.3) |  |  | 90 | (5.2) |  | |  | | 25 | (1.7) |  | |
| ≥60 kg | 61 |  | 4 | (6.9) |  |  | 3 | (5.0) |  | |  | | 1 | (1.0) |  | |
| Diabetes mellitus |  |  |  |  | <0.001 |  |  |  | <0.001 | |  | |  |  | 0.177 | |
| No | 2293 |  | 43 | (2.1) |  |  | 87 | (4.2) |  | |  | | 29 | (1.4) |  | |
| Yes | 373 |  | 21 | (6.1) |  |  | 32 | (9.8) |  | |  | | 8 | (2.3) |  | |
| Previous stroke or TIA |  |  |  |  | <0.001 |  |  |  | <0.001 | |  | |  |  | <0.001 | |
| No | 2645 |  | 57 | (2.4) |  |  | 110 | (4.7) |  | |  | | 33 | (1.3) |  | |
| Yes | 25 |  | 6 | (25.6) |  |  | 8 | (35.5) |  | |  | | 4 | (18.2) |  | |
| Admission diagnosis |  |  |  |  | 0.969 |  |  |  | 0.200 | |  | |  |  | 0.497 | |
| Unstable angina | 408 |  | 9 | (2.3) |  |  | 25 | (6.6) |  | |  | | 6 | (1.5) |  | |
| NSTEMI | 927 |  | 2 | (2.5) |  |  | 37 | (4.7) |  | |  | | 16 | (1.8) |  | |
| STEMI | 1342 |  | 33 | (2.8) |  |  | 57 | (4.7) |  | |  | | 15 | (1.2) |  | |
| Access site |  |  |  |  | 0.009 |  |  |  | 0.130 | |  | |  |  | 0.008 | |
| Radial | 1437 |  | 27 | (2.0) |  |  | 55 | (4.1) |  | |  | | 9 | (0.7) |  | |
| Femoral | 1000 |  | 26 | (2.9) |  |  | 50 | (5.9) |  | |  | | 24 | (2.6) |  | |
| Other | 21 |  | 8 | (7.4) |  |  | 9 | (8.3) |  | |  | | 3 | (2.5) |  | |
| Aspirin, P2Y12 inhibitor and vitamin K antagonist at discharge |  |  |  |  | 0.435 |  |  |  | 0.690 | |  | |  |  | 0.388 | |
| Mono | 24 |  | 1 | (4.5) |  |  | 2 | (8.9) |  | |  | | 1 | (4.3) |  | |
| Double | 2565 |  | 59 | (2.5) |  |  | 113 | (4.9) |  | |  | | 34 | (1.4) |  | |
| Triple | 88 |  | 4 | (4.9) |  |  | 4 | (6.5) |  | |  | | 2 | (2.4) |  | |

*CV* cardiovascular, *MI* myocardial infarction, *NSTEMI* non-ST-elevation myocardial infarction, *STEMI* ST-elevation myocardial infarction, *TIA* transient ischaemic attack, *TIMI* thrombolysis in myocardial infarction, *TVR* percutaneous target vessel revascularisation

* 400 days

† log-rank test

Data represent the number of patients with at least one of the respective outcomes at 1 year, and the corresponding cumulative incidence based on the Kaplan-Meier method (%).
